# Polymorphism of 41 kD Flagellin Gene and Its Human B-Cell Epitope in* Borrelia burgdorferi* Strains of China

**DOI:** 10.1155/2016/1327320

**Published:** 2016-11-15

**Authors:** Huixin Liu, Wei Liu, Xuexia Hou, Lin Zhang, Qin Hao, Kanglin Wan

**Affiliations:** ^1^State Key Laboratory for Infectious Disease Prevention and Control, National Institute for Communicable Disease Control and Prevention, Chinese Center for Disease Control and Prevention, Beijing 102206, China; ^2^Collaborative Innovation Center for Diagnosis and Treatment of Infectious Diseases, Hangzhou 310003, China

## Abstract

The 41 kD flagellin of* Borrelia burgdorferi (B. burgdorferi)* is a major component of periplasmic flagellar filament core and a good candidate for serodiagnosis in early stage of Lyme disease. Here, we chose 89* B. burgdorferi* strains in China, amplified the gene encoding the 41 kD flagellin, and compared the sequences. The results showed that genetic diversity presented in the 41 kD flagellin genes of all 89 strains among the four genotypes of* B. burgdorferi*, especially in the genotype of* B. garinii*. Some specific mutation sites for each genotype of the 41 kD flagellin genes were found, which could be used for genotyping* B. burgdorferi* strains in China. Human B-cell epitope analysis showed that thirteen of 15 nonsynonymous mutations occurred in the epitope region of 41 kD flagellin and thirty of 42 B-cell epitopes were altered due to all 13 nonsynonymous mutations in the epitope region, which may affect the function of the antigen. Nonsynonymous mutations and changed human B-cell epitopes exist in 41 kD flagellin of* B. burgdorferi sensu lato* strains; these changes should be considered in serodiagnosis of Lyme disease.

## 1. Introduction

Lyme disease is a widespread, systemic disease caused by* B. burgdorferi*, which is transmitted to human by* Ixodes ticks* [1, 2]. Its incidence varies between countries, with approximately 65,500 patients annually in Europe [3, 4]. Approximately 30,000 new cases of LD occur in the United States each year [5, 6]. Many studies have confirmed that* B. burgdorferi* is phenotypically and genotypically heterogeneous. To date, 18* B. burgdorferi* genospecies have been described; at least four of these species,* B. burgdorferi sensu stricto (B.b.s.s)*,* B. garinii*,* B. afzelii*, and* B. spielmanii*, are associated with human infection [7–10]. The common causative LD agent in North America is* Borrelia burgdorferi sensu stricto (B.b.s.s).* In Europe and Asia,* Borrelia garinii* and* Borrelia afzelii* are the most abundant species [11, 12]. Clinical manifestations of Lyme disease are diverse, mainly including erythema migrans (EM) skin lesions, acrodermatitis chronica atrophicans, and neurotropic and arthritogenic symptoms. Laboratory evidence of infection, mainly serology, is essential for diagnosis, except in the case of typical EM. Immunological and molecular biological characterization of* B. burgdorferi* has led to the identification of several antigens that may be useful in the development of improved diagnostic methods and vaccines [13].

The 41 kD flagellin is encoded by the gene* flaB* and is a major component of* B. burgdorferi*'s periplasmic flagellar filament core. Recently, a study by Sultan et al. [14] demonstrated that* flaB* mutant of* B. burgdorferi* was nonmotile. They also found that whereas wild-type cells were motile and had a flat-wave morphology,* flaB* mutant cells were nonmotile and rod shaped. Hence, the 41 kD flagellin is critical for optimal survival in ticks and infection of the mammalian host by the arthropod tick vector. Moreover, the first detectable specific immunoglobulin (Ig) M and IgG responses were directed to the 41 kD flagellin in the patients with* B. burgdorferi* infection [15]. It makes this antigen important for serodiagnosis. The 41 kD flagellin is the most human B-cell epitope-harbored antigen containing forty-four B-cell epitopes [16], which suggest that it may play an important role in the immune reaction between* B. burgdorferi* and human B-cells. In order to understand the gene diversity of the 41 kD flagellin of Chinese strains, especially on the human B-cell epitopes, we sequenced and analyzed the 41 kD flagellin gene of 89 strains of four genotypes in China.

## 2. Materials and Methods

### 2.1. Strain Selection

We selected 89* B. burgdorferi* strains which were isolated in Beijing Municipality and 11 provinces and autonomous regions in China (Table 1). These strains were genotyped by multilocus sequence analysis (MLSA) in previous study [17]. 89 strains belonged to four genotypes, which were* B.b.s.s*,* B. garinii*,* B. afzelii*, and* B. valaisiana*. According to the genotypic ratio of* B. burgdorferi* strains in China, we selected 1* B.b.s.s* strain, 67* B. garinii* strains, 16* B. afzelii* strains, and 5* B. valaisiana* strains in this study (Table 2).

### 2.2. Culture and PCR

These strains were cultured in Barbour-Stoenner-Kelly (BSK) medium, collected by centrifuging at 13000 rpm/min, and then heat-inactivated at 100°C. DNA obtained by this method was used as a template for amplifying the gene of 41 kDa flagellin. The nucleotide sequences of the primers used in this study were designed with Primer 5 software according to B31 genome sequence and were as follows: 5′-TTATCATGGAGGAATGATAT-3′ and 5′-ACCCTACTCAAAGCAAACTC-3′. PCR was performed in a total volume of 50 *μ*L. The PCR mix contained 25 *μ*L PCR buffer, 20 pM of each primer, 2.5 mM each of four dNTPs, and 1 U DNA Taq Polymerase (Takara). Amplification was performed for 10 min of initial denaturation at 94°C; 35 cycles under the following conditions: 1 min of denaturation at 94°C, 1 min of annealing at 52°C, and 1 min of extension at 72°C; 10 min of final extension at 72°C. Negative control (reagent only, no DNA) was included when the PCR was performed. The positive control was 300 ng DNA from the B31 of* B.b.s.s* genotype, which was the standard strain in the United States. The presence and size of PCR products were determined by electrophoresis on 1.5% agarose gel in Tris-boric acid-EDTA buffer followed by staining with goldview. We performed all of the PCRs at least twice to validate the reproducibility.

### 2.3. Analytical Methods

The sequences of all PCR products were determined with an ABI 3730xl DNA Analysis. Distances were calculated using the neighbor-joining method. The sequences which contained 46–1011 bp of 41 kD flagellin were compared by MEGA5.10 software [18].

## 3. Results

We amplified the gene encoding the 41 kDa flagellin, obtained PCR products of all 89 strains, and then compared the sequences based on the 41 kDa flagellin gene sequence of B31. As a result, the nucleotide and amino acids sequences of 41 kD flagellin in* B.b.s.s* strain CS4 were exactly identical to B31, whereas there were 133 single nucleotide polymorphisms, consisting of 15 nonsynonymous mutations and 118 synonymous mutations in 41 kDa flagellin genes of the remaining 88 strains (Table 3). As shown in Table 3, except AA position 105 and 279 mutations, 13 nonsynonymous mutations located in epitope region. AA positions 205 and 215 displayed higher polymorphism among 15 nonsynonymous mutations. Strains with other three genotypes,* B. garinii*,* B. afzelii*, and* B. valaisiana*, were almost all changed at AA positions 205 and 215. In addition, AA position 205 had two variants, T(Thr)-A(Ala) and T(Thr)-S(Ser). Moreover, the changes of AA positions 208, 213, and 279 were unique to* B. garinii* strains; AA positions 17, 191, 199, 216, and 230 were unique to* B. afzelii* strains and AA positions 142 and 260 were unique to* B. valaisiana*. The specific mutation sites for each genotype could be used for genotyping Chinese* B. burgdorferi* strains.

The 41 kD flagellin harbored forty-four human B-cell epitopes [16]. Because two of forty-four human B-cell epitopes were not mapped to B31, we analyzed the changes of forty-two epitopes (Table 4). In our study, thirteen of 15 nonsynonymous mutations occurred in the epitope region of 41 kD flagellin and thirty of 42 human B-cell epitopes were altered due to all 13 nonsynonymous mutations in the epitope region. The changed epitopes mainly concentrated in central region of 41 kD flagellin, which was about AA positions 131–266 [19].

**Figure 1:**
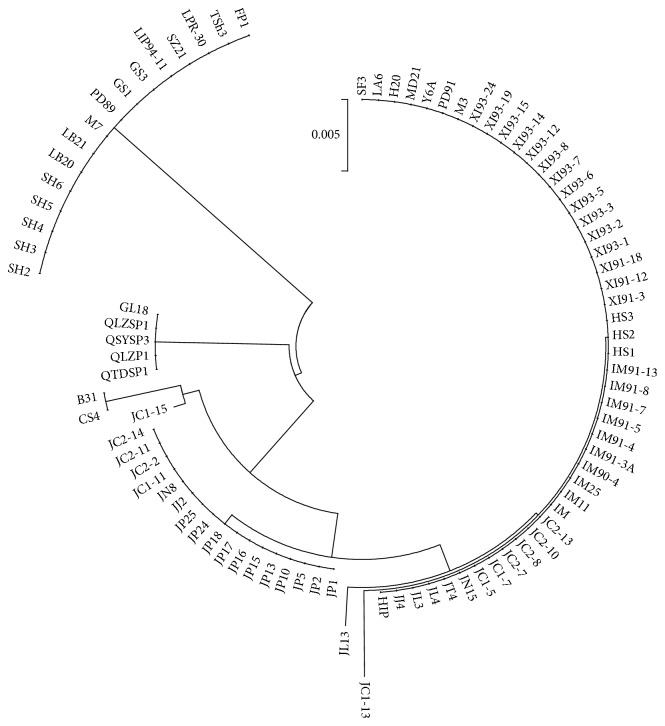
Phylogenetic analysis of 89 strains based on amino acid sequence of 41 kD flagellin.

From Table 3 and Figure 1, we can see that the amino acid sequence of strains within the genotype (16* B. afzelii* and 5* B. valaisiana*) showed almost 100% identity, respectively. Nevertheless, 67* B. garinii* strains showed more sequence variations and could be further divided into two major subgroups. Compared with the same genotype strains, three of 67* B. garinii* strains, JL13, JC1-13, and JC1-15, showed obvious diversity. The only one strain with mutation at AA position 213 was JC1-13. For the mutation site of AA position 224, JC1-15 was the only one of 67* B. garinii* strains.

## 4. Discussion

In this study, we selected 89* B. burgdorferi* strains in China that derived from very large geographical areas and had four MLSA genotyping patterns. Hence, the data provided by them was representative of the genetic diversity that might be presented in China, at least to some extent.

The 41 kD flagellin protein is the predominant component of* B. burgdorferi*'s flagellar filament core [20, 21]. As reported by Namba et al. [22], X-ray fiber diffraction analysis of the secondary flagellin structure indicated that the terminal portions of the protein influence the structure of the filament and are located toward the center of the filament, whereas the central region of the protein is not involved in conformation and is located on the outside of the filament structure. Some studies have demonstrated that this protein and its central region could elicit the immune response to* B. burgdorferi* [15, 23, 24]. In addition, the central region of 41 kD flagellin was specific to* B. burgdorferi* and had no cross-reactive with other* Borrelia* species, such as* Borrelia recurrentis* [24]. However, it has been reported that flagellin genes cloned from several* B. burgdorferi* strains had been shown to be highly conserved [15]. In our study, fifteen nonsynonymous mutations were discovered among 89 strains including four* B. burgdorferi* genotypes; thirteen of 15 nonsynonymous mutations were found in the epitope region, and some of them were unique to each genotype. It is suggested that the sensitivity including 41 kD flagellin of four genotypes may be improved in serodiagnosis assay.

Studies in human pathogenic viruses, bacteria, and protozoa have revealed that the genes encoding antigens tend to be highly variable as a consequence of diversifying selection to evade host immunity [25–28]. A few studies have demonstrated that VlsE and OspC were critical for immune evasion of* B. burgdorferi* [1, 29–32]. The 41 kD flagellin protein containing forty-four human B-cell epitopes may play an important role in humoral immunity between* B. burgdorferi* and human B-cells. We found that the epitope regions of this protein were diverse; 71.43% (30/42) of the B-cell epitopes presented AA changes, which may reflect ongoing immune evasion. The distribution of these nonsynonymous mutations was located mainly in amino acid positions 142–279, consistent with the central region of 41 kD flagellin antigen.

The protein function may be affected by the changed AA's polarity. The changes of AA positions 17, 142, 199, and 205 (T-A) caused the decreasing of hydrophilicity. On the contrary, AA positions 105, 191, 208, and 224 led to the increase of hydrophilicity. The changes of AA's polarity may have a slight impact on the capacity of flagellin penetration, resulting in differences in protein function of each genotype strain.

Based on the diversity of amino acid in 41 kD flagellin, 89* B. burgdorferi* strains were clustered into four groups (Figure 1). These four groups by clustering were consistent with MLSA genotyping pattern. As shown in Figure 1, the amino acid sequences of strains were conserved within each genotype of* B.b.s.s*,* B. afzelii*, and* B. valaisiana*, whereas the strains of* B. garinii* and all strains among four genotypes showed obvious diversity. In particular, the strain of JC1-15 belonging to* B. garinii* was clustered in* Borrelia burgdorferi sensu stricto*. However, further investigations will be needed to determine whether the observed changes are due to immune pressure, other selection pressure, or mere random genetic mutation.

## 5. Conclusion

In China, nonsynonymous mutations and changed human B-cell epitopes exist in 41 kD flagellin of* B. burgdorferi sensu lato* strains that might affect the function of the antigen and reflect ongoing immune evasion. On the other hand, these changes should be considered in serodiagnosis of Lyme disease.

## Figures and Tables

**Table 1:** Distribution of strains in different areas of China.

Areas	Number of isolates
Jilin Province	31
Guangdong Province	2
Inner Mongolia (Neimeng)	11
Shandong Province	1
Liaoning Province	2
Guizhou Province	7
Sichuan Province	6
Heilongjiang Province	9
Xinjiang Uygur Autonomous Region	15
Beijing Municipality	3
Hebei Province	1
Hunan Province	1
Total	89

**Table 2:** Genotypes of 89 Chinese strains by MLSA.

MLSA genotypes	Number of strains
*Borrelia burgdorferi sensu stricto*	1
*Borrelia garinii*	67
*Borrelia afzelii*	16
*Borrelia valaisiana*	5
Total	89

**Table 3:** Base changes (nonsynonymous mutations) in 41 kD flagellin^&^.

Base change	AA position	Number of changed strains				Distribution
		*B.b.s.s*	*B.g*	*B.a*	*B.v*	
GGC(G)-GCC(A)	17	—	—	16	—	ER^#^
GCA(A)-TCA(S)	105	—	—	16	5	NER^#^
TCT(S)-GCT(A)	142	—	—	—	5	ER
AGA(R)-AAA(K)	146	—	—	16	5	ER
GCA(A)-TCA(S)	191	—	—	16	—	ER
TCT(S)-GCT(A)	199	—	—	16	—	ER
ACT(T)-GCT(A)	205	—	66	16	—	ER
ACT(T)-TCT(S)	205	—	—	—	5	ER
GCT(A)-ACT(T)	208	—	66	—	—	ER
GAG(E)-GAC(D)	213	—	1	—	—	ER
GTT(V)-GCT(A)	215	—	66	16	5	ER
CAG(Q)-GAG(E)	216	—	—	16	—	ER
GCA(A)-ACA(T)	224	—	1	16	—	ER
TCT(S)-ACT(T)	230	—	—	16	—	ER
ATA(I)-GTG(V)	260	—	—	—	5	ER
AAT(N)-GAT(D)	279	—	50	—	—	NER

^&^The CDS of 41 kD flagellin of *Borrelia burgdorferi sensu stricto* B31 has been used as the reference sequence.

^#^ER: epitope region; ^#^NER: nonepitope region.

**Table 4:** Amino acid changes of the B-cell epitopes included in 41 kD flagellin antigen.

IEDB-ID	Epitopes	Base change
26607	IINHNTSAINASRNN**G**INAANLSKTQEKLSSGYRIN	GGC(G)-GCC(A)
27732	INRIADQAQY	No change
27733	INRIADQAQYNQMHMLSNKSA**S**QNV**R**TAEELGMQPAKI	TCT(S)-GCT(A), AGA(R)-AAA(K)
54118	RIADQAQYNQ	No change
752	ADQAQYNQMH	No change
50350	QAQYNQMHML	No change
53012	QYNQMHMLSN	No change
45589	NQMHMLSNKS	No change
41667	MHMLSNKSA**S**	TCT(S)-GCT(A)
42062	MLSNKSA**S**QN	TCT(S)-GCT(A)
59856	SNKSA**S**QNV**R**	TCT(S)-GCT(A), AGA(R)-AAA(K)
7895	DEAIAVNIY**A**ANVANLF**S**GEGAQ**T**AQ**A**APVQ**E**G**V**Q**Q**E	GCA(A)-TCA(S), TCT(S)-GCT(A), ACT(T)-G(T)CT(AS), GCT(A)-ACT(T) GAG(E)-GAC(D), GTT(V)-GCT(A) CAG(Q)-GAG(E)
35977	LF**S**GEGAQ**T**AQ**A**APVQ**E**G**V**Q**Q**EGAQQP**A**PATAP**S**QGGVNSPVNVT	TCT(S)-GCT(A), ACT(T)-G(T)CT(A S)GCT(A)-ACT(T), GAG(E)-GAC(D), GTT(V)-GCT(A), CAG(Q)-GAG(E), GCA(A)-ACA(T), TCT(S)-ACT(T)
3341	ANLF**S**GEGAQ**T**AQ	TCT(S)-GCT(A), ACT(T)-G(T)CT(A S)
58007	**S**GEGAQ**T**AQ**A**APV	TCT(S)-GCT(A), ACT(T)-G(T)CT(A S), GCT(A)-ACT(T)
52474	Q**T**AQ**A**APVQ**E**G**V**Q	ACT(T)-G(T)CT(A S), GCT(A)-ACT(T) GAG(E)-GAC(D), GTT(V)-GCT(A)
62967	**T**AQ**A**APVQ**E**G	ACT(T)-G(T)CT(A S), GCT(A)-ACT(T) GAG(E)-GAC(D)
62968	**T**AQ**A**APVQ**E**G**V**Q**Q**EGAQQP**A**PA	ACT(T)-G(T)CT(A S), GCT(A)-ACT(T) GAG(E)-GAC(D), GTT(V)-GCT(A) CAG(Q)-GAG(E), GCA(A)-ACA(T)
50213	Q**A**APVQ**E**G**V**Q	GCT(A)-ACT(T), GAG(E)-GAC(D) GTT(V)-GCT(A)
379	**A**APVQ**E**G**V**Q**Q**EGA	GCT(A)-ACT(T), GAG(E)-GAC(D) GTT(V)-GCT(A), CAG(Q)-GAG(E)
3893	APVQ**E**G**V**Q**Q**E	GAG(E)-GAC(D), GTT(V)-GCT(A) CAG(Q)-GAG(E)
70549	VQ**E**G**V**Q**Q**EGA	GAG(E)-GAC(D), GTT(V)-GCT(A) CAG(Q)-GAG(E)
70550	VQ**E**G**V**Q**Q**EGAQQP	GAG(E)-GAC(D), GTT(V)-GCT(A) CAG(Q)-GAG(E)
50610	Q**E**G**V**Q**Q**EGAQQP**A**P	GAG(E)-GAC(D), GTT(V)-GCT(A) CAG(Q)-GAG(E), GCA(A)-ACA(T)
12306	**E**G**V**Q**Q**EGAQQ	GAG(E)-GAC(D), GTT(V)-GCT(A), CAG(Q)-GAG(E)
12307	**E**G**V**Q**Q**EGAQQP**A**	GAG(E)-GAC(D), GTT(V)-GCT(A) CAG(Q)-GAG(E), GCA(A)-ACA(T)
70628	**V**Q**Q**EGAQQP**A**	GTT(V)-GCT(A), CAG(Q)-GAG(E), GCA(A)-ACA(T)
52010	Q**Q**EGAQQP**A**PATA	CAG(Q)-GAG(E), GCA(A)-ACA(T)
50597	**Q**EGAQQP**A**PA	CAG(Q)-GAG(E), GCA(A)-ACA(T)
51693	QP**A**PATAP**S**QGGV	GCA(A)-ACA(T), TCT(S)-ACT(T)
3820	AP**S**QGGVNSPVNV	TCT(S)-ACT(T)
3821	AP**S**QGGVNSPVNVTTTVDANTSLAKIENAIRM**I**SDQRANLGAFQNR	TCT(S)-ACT(T), ATA(I)-GTG(V)
25890	IENAIRM**I**SD	ATA(I)-GTG(V)
43169	NAIRM**I**SDQR	ATA(I)-GTG(V)
28340	IRM**I**SDQRAN	ATA(I)-GTG(V)
41672	M**I**SDQRANLG	ATA(I)-GTG(V)
57265	SDQRANLGAF	No change
52186	QRANLGAFQN	No change
3342	ANLGAFQNRL	No change
36055	LGAFQNRLES	No change
1379	AFQNRLESIK	No change
65265	TMTDEVVAATTNSILTQSAMAMIAQANQVPQYVLSLLR	No change

Note: letters in bold font in the table represent the mutated amino acids in epitopes.
